# Effectiveness of ChAdOx1 nCoV-19 and BBIBP-CorV vaccines against COVID-19-associated hospitalisation and death in the Seychelles infected adult population

**DOI:** 10.1371/journal.pone.0299747

**Published:** 2024-04-05

**Authors:** Sylvie Nadine Theresa Pool, Emelyn Helen Shroff, Agnes Chetty, Lara Lewis, Yende-Zuma Nonhlanhla, Salim S. Abdool Karim

**Affiliations:** 1 Health Care Agency, Ministry of Health, Mont Fleuri, Mahe Seychelles; 2 Public Health Authority, Ministry of Health, Mont Fleuri, Mahe Seychelles; 3 Ministry of Health, Mont Fleuri, Mahe Seychelles; 4 Centre for the Aids Programme of Research in South Africa, University of KwaZulu-Natal, Durban, South Africa; Sheikh Hasina National Institute of Burn & Plastic Surgery, BANGLADESH

## Abstract

**Background:**

The Seychelles COVID-19 vaccination campaign was initiated using two different vaccines during the first wave of the pandemic in 2021. This observational study estimated vaccine effectiveness against severe outcomes (hospitalisation and/or death) from individuals infected with COVID-19 in the Seychelles adult population during Beta and Delta variant transmission.

**Methods:**

This nationwide retrospective cohort study included all Seychellois residents aged ≥ 18 years who tested positive by RT-PCR or rapid antigen test for COVID-19 between January 25, 2021, and June 30, 2021. We measured the relative risk (RR) of laboratory-confirmed SARS-CoV-2 hospitalisation and/or death among individuals partially or fully vaccinated with ChAdOx1 nCoV-19 (SII Covishield) or BBIBP-CorV (Sinopharm) vaccines compared to unvaccinated individuals using modified Poisson regression. Controlling for age, gender and calendar month, vaccine effectiveness was estimated as 1-RR ≥14 days after the first dose and ≥7 days after the second dose for each available vaccine versus an unvaccinated control group.

**Results:**

A total of 12,326 COVID-19 infections were reported in adult Seychellois residents between January 25, 2021, and June 30, 2021. Of these, 1,287 individuals received one dose of either BBIBP-CorV (Sinopharm) or ChAdOx1-nCoV-19 (SII Covishield) vaccine, and 5,225 individuals received two doses. Estimated adjusted effectiveness of two doses of either Sinopharm or SII Covishield was high, at 70% (95% CI 58%–78%) and 71% (95% CI 62%–78%) respectively. Sinopharm maintained high levels of protection against severe outcomes in partially vaccinated individuals at 61% (95% CI 36%–76%), while the effectiveness of one dose of SII Covishield was low at 29% (95% CI 1%–49%).

**Conclusions:**

This observational study demonstrated high levels of protection of two doses of two vaccine types against severe outcomes of COVID-19 during the first wave of the pandemic driven by Beta (B.1.351) and Delta (B.1.617.2) variant predominance. One dose of ChAdOx1-nCoV-19 (Covishield SII) was found to be inadequate in protecting the general adult population against hospitalisation and/or death from COVID-19.

## Introduction

As of January 13, 2023, more than half of the population of Seychelles have been infected with COVID-19 (i.e., over 50,732 confirmed cases), with a total of 172 deaths. As a Small Island Developing State (SIDS) already faced with challenges by virtue of the country’s small economy of scale, relative geographical isolation from key markets, dependence on tourism and high vulnerabilities to external shocks and climate change, the COVID-19 pandemic has had a disproportionate socioeconomic impact and has placed unparalleled pressure on the national healthcare system.

Alongside non-pharmaceutical interventions (NPIs), COVID-19 vaccines have been hailed as a critical tool in the public health arsenal against SARS-CoV-2 and essential to the recovery effort. Several studies have shown COVID-19 vaccines to be highly efficacious against symptomatic disease [1–6]. However, challenges in delivering vaccines in real-world settings coupled with the emergence of new variants, have meant that levels of efficacy in randomised controlled trials have not necessarily translated into comparable levels of effectiveness in practice. Moreover, questions remain over population differences in vaccine-mediated immune response, particularly in more ethnically diverse populations [7–9].

Seychelles launched its COVID-19 vaccination campaign on January 10, 2021, with the inactivated vaccine BBIBP-CorV (Sinopharm) closely followed by the ChAdOx1 nCoV-19 (SII Covishield) viral vector vaccine. The viral vector vaccine, rAd26-rAd5 (Sputnik V) and mRNA vaccine, BNT162b2 (Pfizer-BioNTech), were introduced in May and September 2021, respectively; the latter aimed primarily at adolescents aged 12–17 years. While the dosing schedule for BBIBP-CorV (Sinopharm) was maintained at 3 weeks, populations eligible for the ChAdOx1 nCoV-19 (SII Covishield) vaccine received their second dose at an extended dosing interval of 6–8 weeks; as evidenced by findings from phase 3 trials demonstrating higher antibody levels and greater vaccine efficacy with longer interdose intervals [10,11].

Several effectiveness studies of non-replicative vaccine platforms have been conducted globally, but less is known about the effectiveness of these vaccines in African countries and SIDS, such as the Seychelles. Moreover, few studies exist on the effectiveness of BBIBP-CorV (Sinopharm), particularly against variants of concern. The importance of understanding the real-world performance of these vaccines is highlighted by the notable differences in vaccine effectiveness (VE) observed across countries. In a cohort study from the United Arab Emirates (UAE) and a similar study conducted in healthcare workers in Peru, a single dose of BBIBP-CorV (Sinopharm) was shown to offer no protection against COVID-19-related death [12,13]. In contrast, a study done in Argentina estimated effectiveness of one dose of BBIBP-CorV (Sinopharm) at over 70% against COVID-19-related mortality.(8) In another study from Hungary, VE estimates for two doses of Sinopharm against COVID-19-related hospitalisation were found to be significantly lower (estimated at 53.8%) than those presented in a similar study from China that reported an estimated VE of 100% effectiveness against the same outcome, for the same circulating Delta variant of concern (VOC) [14,15].

The Seychelles context offers a unique opportunity to evaluate the real-world performance of two different COVID-19 vaccines in a single multi-ethnic population. The aim of this study is to estimate the effectiveness of namely BBIBP-CorV (Sinopharm) and ChAdOx1 nCoV-19 (SII Covishield) against hospitalisation and/or death due to COVID-19 in the Seychelles infected adult population.

## Materials and methods

### Setting

At the start of the COVID-19 vaccination campaign, priority for vaccination with BBIBP-CorV (Sinopharm) was given to essential workers thought to be at increased risk of exposure to COVID-19 and individuals in the 18–59 age group without existing comorbidities (in adherence with Sinopharm manufacturer guidance). Seychellois citizens and permanent residents were prioritized for the initially limited (donated) supply of BBIBP-CorV (Sinopharm) doses. Vaccination of more vulnerable subgroups i.e. older age groups of 60 years and older and those between the ages of 18–59 years with existing comorbidities, began in late January 2021 with the ChAdOx1 nCoV-19 (SII Covishield) viral vector vaccine based on phase 3 trials and post-marketing studies demonstrating its effectiveness in these subgroups. By the end of February 2021, administration of the ChAdOx1 nCoV-19 (SII Covishield) had been extended to all other adult age groups (18 years and above) regardless of comorbidities, in an effort to maximise adult population vaccine coverage.

Despite relatively high vaccination coverage rates by the first quarter of 2021, the incidence of COVID-19 cases rose sharply from February 2021 peaking at over 2000 cases per week in early May 2021, signalling the first ‘wave’ of the pandemic. In addition, SARS-CoV-2 variants of concern (VOC) namely, Alpha (B.1.1.7), Beta (B.1.351) and Delta (B.1.617.2) variants, were detected locally. Available sequencing data, points to both Beta (B.1.351), and by mid-May 2021, Delta (B.1.617.2) variants being significant drivers of the surge in April–June 2021 (S1 Fig).

By March 2021 testing efforts had also been scaled up to provide rapid antigen and RT-PCR testing freely at all health facilities, both public and private. Triage testing stations were set-up at all health facility entry points, to identify potentially infected individuals for timely isolation, contact tracing and clinical management with the goal of breaking the chain of transmission and minimising morbidity and mortality. Up until August 2021 all positive cases not meeting home isolation criteria were isolated in Government isolation facilities and an adapted clinical scoring system was employed to determine need for isolation in COVID-19-designated hospitals, whereby the elderly, those with existing comorbidities, pregnant women and the unvaccinated were prioritised for hospital admission.

### Study design

This nationwide, retrospective cohort study assessed the effectiveness of the inactivated vaccine, BBIBP-CorV (Sinopharm) and the viral vector vaccine, ChAdOx1 nCoV-19 (SII Covishield) against COVID-19-related hospitalization and/or death post-COVID-19 diagnosis. The study population included all Seychelles residents aged 18 or older that tested positive by RT-PCR or rapid antigen test, for COVID-19 between 25 January 2021 and 30 June 2021.

The start date of the study was set to start on 25^th^ January 2021, 14 days after the first COVID-19 vaccination was administered in Seychelles. BBIBP-CorV (Sinopharm) was first administered on 10 January 2021 and ChAdOx1 nCoV-19 (SII Covishield) on 28 January 2021.

### Data sources and management

The number of vaccinated and unvaccinated individuals in the Seychelles from the start of the vaccination roll-out to 30 June 2021 was calculated using the ‘Vaccination Administration Management System’ (VAMS). Data on COVID-19 positive cases were collected and combined from four national databases linked by individual national identification numbers. COVID-19 positive test results were obtained from the Disease Surveillance and Response Unit (DSRU) positive case database as well as the Seychelles Public Health Laboratory (SPHL) RT-PCR (Flu) database. These compiled cases offer a comprehensive depiction of the total COVID-19 case load within the country through the specified timeframe of the study. Clinical data on hospitalisation and death were obtained from digitalised hospital admissions records and vaccination data extracted from the ‘Vaccination Administration Management System’ (VAMS). The data was extracted on 26^th^ July 2021 for the study period of 25^th^ January 2021 and 30^th^ June 2021. The combined dataset included the date of COVID-19 diagnosis, vaccine status at the time of diagnosis, vaccine type and date(s) of vaccinations (if applicable), age, gender, as well as date of COVID-19 related hospitalisation and/or death (if applicable). Once linked, all personal identifiers were removed from the database before analysis.

Individuals missing data on the aforementioned variables were excluded from analysis. Where the vaccination status (both in terms of vaccine type and date of administration) could not be corroborated with the VAMS database due to incomplete records in the DSRU database, individuals were excluded. Individuals vaccinated with 2 different vaccine types for their first and second doses were also excluded. Individuals with infections occurring within 14 days of their first vaccination dose were also excluded from analysis. This study was approved by the Health and Research Ethics Committee of the Public Health Authority of the Ministry of Health Seychelles, proposal number 2105. This study was conducted in accordance with the relevant guidelines and regulations. This retrospective study used deidentified medical data of adult population aged 18–65 years and did not require individual participant consent. No data from minors were included in this study.

### Variables

The primary endpoint was defined as a binary variable capturing COVID-19-related hospitalisation and/or death. The primary exposure of interest was vaccination status at time of COVID-19 infection. Individuals were defined as being partially vaccinated at diagnosis if they tested COVID-19 positive 14 or more days after their first vaccination dose and less than 7 days after their second vaccination dose, as fully vaccinated at diagnosis if they tested COVID-19 positive 7 or more days after their second vaccination dose and as unvaccinated at diagnosis otherwise. Repeat infections that occurred within 3 months of each other were assumed to be the same infection and the earliest positive test date was used as the infection date. Re-infections were included in the analysis although there were few. Vaccination status was also split by vaccine type resulting in the vaccination exposure variable having 5 categories: (i) unvaccinated, (ii) partially vaccinated with BBIBP-CorV (Sinopharm), (iii) fully vaccinated with BBIBP-CorV (Sinopharm), (iv) partially vaccinated with ChAdOx1 nCoV-19 (SII Covishield) and (v) fully vaccinated with ChAdOx1 nCoV-19 (SII Covishield).

### Statistical analysis

Characteristics of the sample were summarised using medians and interquartile range (IQR) for continuous variables and frequencies and percentages for categorical ones. Characteristics of vaccination exposure groups were compared using the Chi-square test (categorical variables) and the Wilcoxon rank sum test (continuous variables). The cumulative incidence of COVID-19 for each exposure group was approximated using the ratio of the total number of infections in the group over the mean number of individuals in the group between 1 February 2021 and 30 June 2021.

Modified Poisson regression was used to estimate relative risk (RR) of COVID-19-related hospitalisation and/or death post COVID-19 diagnosis by vaccination status, adjusting for age, gender, and calendar month. Calendar month was included to adjust for changes in testing practices, non-pharmaceutical interventions, COVID-19 treatment approaches and dominant variants of concern over time. As it was hypothesized that the effectiveness of a vaccine could vary with the age of an individual or with time, interaction terms between vaccine status and age and calendar month respectively were included in the model if found to significantly improve model fit, where model fit was assessed using the Quasilikelihood under the Independence model Criterion (QIC) statistic. Vaccine effectiveness (VE) was estimated as 1 –RR.

A time-to-event analysis was not adopted for the primary analysis as the time to COVID-19-related hospitalisation/ death was observed to be short and similar for most infected individuals. It was however performed as a secondary analysis using Cox proportional hazards models and censoring individuals at 30 days post COVID-19 diagnosis if a hospitalisation or death had not occurred in this window. VE was estimated as 1 minus the hazard ratio. A sensitivity analysis was also performed in which all individuals missing vaccination status were assumed to be unvaccinated. All analyses were conducted in SAS version 9.4 and employed a statistical significance level of 0.05.

### Results

**Fig 1:**
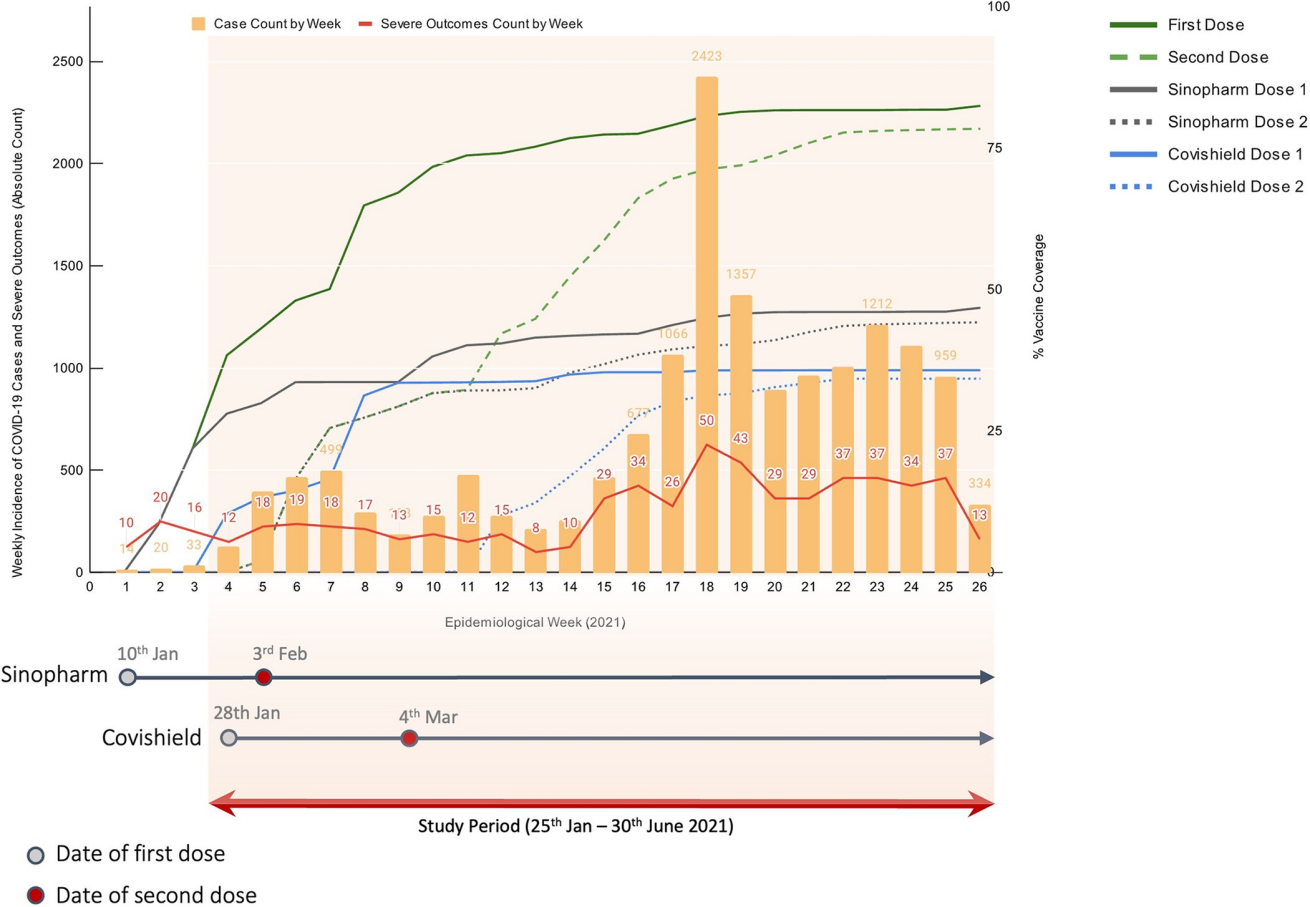
Weekly cases of confirmed COVID-19 infections and severe outcomes (hospitalization and/or deaths), vaccine coverage (%) and launched dates (first and second doses).

Fig 1 shows the weekly incidence of COVID-19 infections and confirmed COVID-19 related hospitalisations and/or deaths, vaccine coverage (as a percentage of the total eligible population) by week and the introduction of BBIBP-CorV (Sinopharm) and ChAdOx1 nCoV-19 (SII Covishield) with the date of their respective second doses. By the end of June 2021, over 80% (N = 67,629) of the Seychelles adult population had received at least one dose of either the BBIBP-CorV (Sinopharm) or ChAdOx1 nCoV-19 (SII Covishield) vaccine.

A total of 15,954 COVID-19 infections were recorded in the Seychelles between 1 January 2021 and 30 June 2021. The number of infections peaked in May 2021 and remained high throughout June 2021.

**Fig 2:**
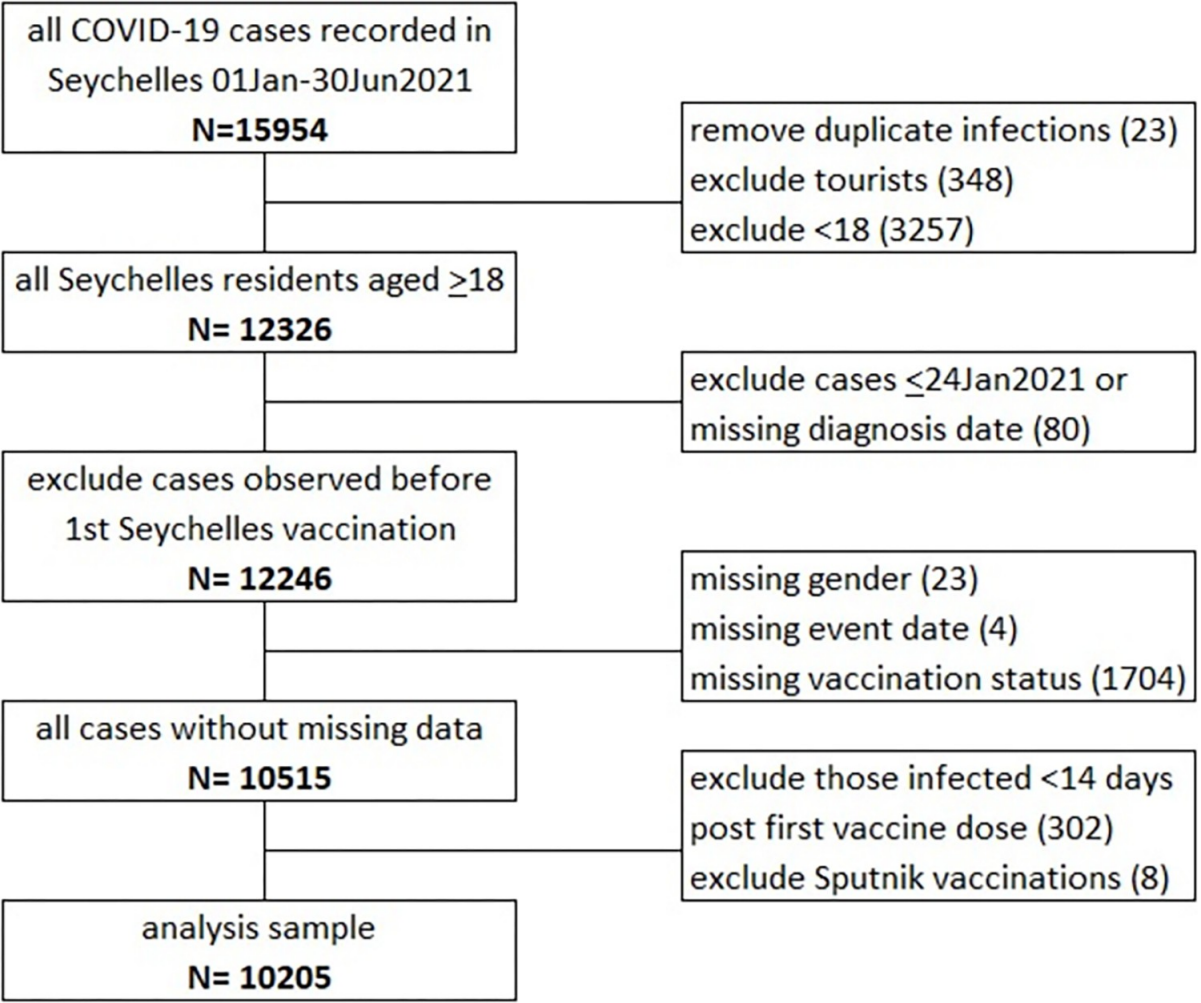
Study profile- Flow chart of creation of analysis samples.

Of the 15,954 infections, 12,326 occurred among adult residents of the Seychelles (Fig 2). A total of 12,246 were known to have occurred more than 14 days after the first vaccination was administered in the Seychelles. Of the 12,246, 1704 (14%) had to be excluded from the analysis because of incomplete or inconsistent vaccination data. Another 27 (1%) were missing data on gender or hospitalization/death data. A further 302 (2%) were excluded because their infection was identified 14 days within receiving their first vaccination dose and 8 (<0.1%) were excluded because they received a vaccination that was neither the BBIBP-CorV (Sinopharm) or ChAdOx1 nCoV-19 (SII Covishield) vaccine. The remaining sample comprised of 10,205 individuals.

Among the 10,205 individuals included in the analysis sample, 3,693 were not vaccinated at the time of diagnosis, 581 and 706 were partially vaccinated with ChAdOx1 nCoV-19 (SII Covishield) or BBIBP-CorV (Sinopharm), respectively, and 1315 and 3910 were fully vaccinated with ChAdOx1 nCoV-19 (SII Covishield) or BBIBP-CorV (Sinopharm) respectively (Table 1). The median (IQR) age of the sample was 37(28–49). The ages of those partially or fully vaccinated with ChAdOx1 nCoV-19 (SII Covishield) were greater than those in other exposure groups owing to the prioritization of ChAdOx1 nCoV-19 (SII Covishield) for older age groups during the observation period 47(33–62) compared to 36(27–46), p<0.001. A higher proportion of unvaccinated individuals were men compared to vaccinated individuals (48.4% versus 43.6%, p<0.001). Overall, 402 (3.9%) individuals were hospitalised following diagnosis with a median (IQR) time to hospitalisation of 1(0–4) days after diagnosis. Time to hospitalisation was similar across all exposure groups. Seventy (0.7%) individuals died following diagnosis, with a median (IQR) time to death from diagnosis of 1(0–5) days. Not all individuals who died were hospitalised prior to death. The characteristics of those who had missing vaccination status and/or date and were excluded from the sample were compared to those in the analysis sample (S1 Table). The proportion of women and those who died were lower in the sample with missing vaccination status and/or date.

**Table 1:** Sample description of adult Seychelles residents who tested positive for COVID-19 from 24 January to 30 June 2021, stratified by vaccination status at time of diagnosis.

		Un-vaccinated	Covishield		Sinopharm		Total
			Full	Partial	Full	Partial	
N		3693	1315	581	3910	706	10205
**Cumulative incidence over 5-month period** *		23%	10%	5%	16%	8%	14%
**Age**	**median (IQR)**	34(25–44)	47(34–62)	46(32–62)	38(29–47)	38(29–48)	37(28–49)
	**18–19, %(n)**	8.5(315)	2.3(30)	1.7(10)	2.2(85)	1.3(9)	4.4(449)
	**20–29, %(n)**	29.9(1104)	15.4(202)	18.4(107)	24.5(958)	26.1(184)	25(2555)
	**30–39, %(n)**	25.8(954)	17.9(235)	20.1(117)	28(1096)	27.8(196)	25.5(2598)
	**40–49, %(n)**	19.1(704)	18.4(242)	15.7(91)	25.1(983)	22(155)	21.3(2175)
	**50–59, %(n)**	9.5(352)	14.8(195)	13.1(76)	18.3(717)	20.4(144)	14.5(1484)
	**60–69, %(n)**	3.7(137)	20.2(265)	18.9(110)	1.8(69)	2(14)	5.8(595)
	**70+, %(n)**	3.4(127)	11.1(146)	12(70)	0.1(2)	0.6(4)	3.4(349)
**Gender**	**Female, %(n)**	51.6(1905)	54.6(718)	50.9(296)	57.7(2257)	57.4(405)	54.7(5581)
	**Male, %(n)**	48.4(1788)	45.4(597)	49.1(285)	42.3(1653)	42.6(301)	45.3(4624)
**Hospitalization**	**%(n)**	5.9(217)	3.9(51)	8.8(51)	1.7(65)	2.5(18)	3.9(402)
	**days from diagnosis to hospitalisation, median (IQR)**	1(0–4)	2(0–4)	1(0–4)	2(0–5)	1(0–3)	1(0–4)
**Death**	**%(n)**	1.5(57)	0.6(8)	0.3(2)	0.1(2)	0.1(1)	0.7(70)
	**days from diagnosis to death, median (IQR)**	1(0–4)	1.5(0–8)	0.5(0–1)	7(6–8)	0(0–0)	1(0–5)

*Estimated as the number of infections over the average number at risk between 01Feb2021 and 30Jun2021.

#### Relative risk and vaccine effectiveness

The estimates of the relative risk of being hospitalised or dying due to COVID-19 are presented in Table 2. When comparing the crude proportions, the risk of hospitalisation and death was lower in those fully vaccinated with either vaccine and those partially vaccinated with BBIBP-CorV (Sinopharm) compared to those who were unvaccinated. However, the risk of hospitalisation and death was higher in those partially vaccinated with ChAdOx1 nCoV-19 (SII Covishield) compared to those who were unvaccinated. The multivariable model adjusted for age, gender, vaccine status and month of infection. Planned interaction terms were not included in the model as they did not significantly improve the model fit. After adjustment for age, gender and month of infection, the relative risk of hospitalisation and death was significantly lower in all vaccine exposure groups compared to the unvaccinated group.

**Table 2:** Relative risk (RR) of COVID-19 related hospitalization/death.

		%(n) events	RR	95% CI	Adjusted RR	95% CI
**Age in years**	18–29	1.5(45)	1		1	
	30–39	2.4(63)	1.62	1.1–2.37	1.77	1.21–2.6
	40–49	3.3(71)	2.18	1.5–3.17	2.56	1.76–3.72
	50–59	5.7(84)	3.78	2.63–5.43	4.93	3.41–7.1
	60+	16.9(160)	11.31	8.13–15.75	12.84	9.1–18.11
**Gender**	Female	4(221)	1		1	
	Male	4.4(202)	1.1	0.91–1.34	1.1	0.9–1.33
**Vaccine type**	no vaccine	6.4(237)	1		1	
	Covishield full	3.9(51)	0.6	0.45–0.82	0.3	0.22–0.42
	Covishield partial	9(52)	1.39	1.03–1.88	0.71	0.51–0.99
	Sinopharm full	1.7(65)	0.26	0.2–0.34	0.29	0.22–0.38
	Sinopharm partial	2.5(18)	0.4	0.25–0.64	0.39	0.24–0.64
**Month of infection**	January/February 2021	6.2(57)	1		1	
	March 2021	5.2(50)	0.85	0.58–1.24	0.74	0.5–1.1
	April 2021	5.5(88)	0.88	0.63–1.23	1.09	0.77–1.53
	May 2021	3(117)	0.48	0.35–0.66	0.71	0.51–0.99
	June 2021	3.9(111)	0.63	0.46–0.87	0.9	0.64–1.27

The VE of BBIBP-CorV (Sinopharm) and ChAdOx1 nCoV-19 (SII Covishield) among fully vaccinated individuals were comparable and estimated to be 0.71 (95% confidence interval [CI]: 0.62–0.78) and 0.7 (95% CI: 0.58–0.78) respectively (Table 3). The VE of BBIBP-CorV (Sinopharm) and ChAdOx1 nCoV-19 (SII Covishield) among partially vaccinated individuals were 0.61 (95% CI: 0.36–0.76) and 0.29 (95% CI: 0.01–0.49) respectively. While no differences were observed in the VE of BBIBP-CorV (Sinopharm) and ChAdOx1 nCoV-19 (SII Covishield) vaccines among fully vaccinated individuals, the VE of BBIBP-CorV (Sinopharm) was significantly higher than that of ChAdOx1 nCoV-19 (SII Covishield) among partially vaccinated individuals (p = 0.0384). Estimates of VE using a Cox proportional hazards model produced similar results (S2 Table). The sensitivity analysis produced lower VE estimates against hospitalisation and/or death among fully and partially vaccinated individuals for both vaccines; however, all except partial vaccination with ChAdOx1 nCoV-19 (SII Covishield) still showed a protective effect, Table 3.

**Table 3:** Vaccine effectiveness (VE) against hospitalization/death by vaccine type.

Vaccine type	VE	95% CI
Covishield full	0.7	0.58–0.78
Covishield partial	0.29	0.01–0.49
Sinopharm full	0.71	0.62–0.78
Sinopharm partial	0.61	0.36–0.76

## Discussion

This nationwide observational study provides real-world vaccine effectiveness estimates for two vaccine types against COVID-19-related hospitalisation and death within the context of a Beta (B.1.351) and Delta (B.1.617.2) variant outbreak in Seychelles. Our study found that after adjusting for age, gender, and calendar month, a two-dose primary series of either BBIBP-CorV (Sinopharm) or ChAdOx1 nCoV-19 (SII Covishield) vaccines were equally and highly effective at preventing hospitalisation and death from COVID-19, at an estimated adjusted VE of 71% and 70%, respectively. However, effectiveness against the same outcome following partial vaccination with the ChAdOx1 nCoV-19 (SII Covishield) vaccine was significantly lower, at 29%, compared to BBIBP-CorV (Sinopharm), at 61%.

Approximately **328** severe events from COVID-19 (hospitalisations or deaths) were averted by the vaccination campaign over the study period.

Our findings showed lower effectiveness in fully vaccinated individuals with BBIBP-CorV (Sinopharm) than was reported in phase 3 trials conducted in the Middle East that demonstrated an efficacy of 100% against severe COVID-19 cases (as a composite of severe cases and deaths) [4]. However, it should be noted that, efficacy against this secondary endpoint was based upon only two severe cases within the control group, and as cautioned by authors, were too few to be reliable. Furthermore, the trial involved healthy, young (mean age 36.2) and, predominantly, male participants followed-up over a short period of 77 days [4]. By contrast, our results reflect a real-world context with the inclusion of more diverse and high-risk populations followed over a longer duration (of 5 months) during a period of emerging VOC. These, together with programmatic factors, such as potential challenges with cold-chain management and logistics, may well have contributed to the lower effectiveness rates observed in this study.

VE estimates for full immunisation with BBIBP-CorV (Sinopharm) were also notably lower than those across the majority of observational studies. In all but two studies, BBIBP-CorV (Sinopharm) VE estimates against severe outcomes ranged from 75% to 100%, [8,9,12–14,16–20], allowing for the heterogeneity of studies. Studies with more modest VE estimates took account of the effect of Delta variant transmission. An ambispective cohort study assessing VE of BBIBP-CorV (Sinopharm) against COVID-19-related ICU admission among healthcare workers across Egypt, and a study from Hungary (HUN-VE 3) estimating effectiveness of six different vaccine types, including BBIBP-CorV (Sinopharm) and ChAdOx1 nCoV-2 against COVID-19-related hospitalisation and death, both reported more modest BBIBP-CorV (Sinopharm) VE estimates during a period of delta variant transmission, compared to other studies [15–20]. These were reported as an overall adjusted VE of 65% and 45.7% against hospitalisation in studies from Egypt and Hungary, respectively, and 58.6% against COVID-19-related death in the study from Hungary [15,21]. To our knowledge, our study is the third to report on BBIBP-CorV (Sinopharm) effectiveness data within the context of a delta variant transmission. The more blunted results observed, may similarly reflect the impact of the delta variant on BBIBP-CorV (Sinopharm) effectiveness, though this was not directly tested. Nevertheless, our VE estimate (of 71%) remains higher than is reported in previous studies from Egypt and Hungary, possibly owing to the presence of co-circulating Beta variant in Seychelles, dominant in January to Mid-May 2021, to which 2 doses of inactivated vaccine appears to confer better protection [12]. VE estimates for BBIBP-CorV (Sinopharm) in partially vaccinated individuals showed high vaccine effectiveness (61%), but observational studies were too few for reliable comparison.

Similar to BBIBP-CorV (Sinopharm), our findings showed lower effectiveness of two doses of ChAdOx1 nCoV-19 (SII Covishield) compared to the reported efficacy estimates in phase 3 trials. In a pooled analysis of data from four randomised controlled trials (RCT) in the United Kingdom, Brazil and South Africa, efficacy of ChAdOx1 nCoV-19 against hospitalisation or death was reported at 100% [2]. Similarly, in a multinational RCT conducted in the United States, Peru and Chile, the estimated vaccine efficacy against hospitalisation, as an exploratory endpoint, was found to be 94.2% [10]. The difference observed between our results and those of phase 3 trials may again represent the confounding effects of programmatic challenges in real-world settings. Moreover, phase 3 trials were conducted during a period of predominantly wild-type virus transmission and are thus, unlikely to reflect the ‘real’ efficacy under emerging VOC.

Our VE estimates for ChAdOx1 nCoV-19 (SII Covishield) are consistent with other observational studies only insofar as demonstrating an increase in vaccine effectiveness after the second dose (i.e. from 29% to 70% effectiveness). VE estimates after one and two doses of ChAdOx1 nCoV-19 were otherwise lower than reported in other observational studies conducted during Delta and Beta variant predominance [8,15,18,22–25]. A province-wide study from Canada estimated effectiveness of one dose of ChAdOx1 nCoV-19 against severe outcomes caused by the Beta and Delta VOC at 61% and 91%, respectively [22]. Whilst, estimates for two doses of the vaccine were found to be higher, at 90–91% [22]. In another study from India, conducted during a period of Delta dominance, ChAdOx1 nCoV-19 (SII Covishield) was found to be 86% effective against severe outcomes, 14 days after the first dose of the vaccine, increasing to 99% effectiveness after two doses [23]. It is important to recognise that both these studies included younger age groups (mean age 38–44.4 years) in their study populations [22,23] compared to older individuals featured in our study–reflecting the prioritisation of ChAdOx1 nCoV-19 (SII Covishield) in the population aged 60 years and older in Seychelles.

Nevertheless, large-scale population-based studies have shown that ChAdOx1 nCoV-19 remains highly protective in older age groups [15,24,25]. A nationwide retrospective cohort study from Hungary conducted during the Delta transmission, estimated a VE of 73.8% against hospitalisation and 84.8% against mortality in the 65 and over age group, between 14 and 120 days after the second dose of ChAdOx1 nCoV-19 [15]. Similarly, in a large prospective study of over 1.3 million older adults (mean age of 65.0 years) in Scotland, a single dose of ChAdOx1 nCoV-19 vaccine was shown to be 88% effective in protecting against hospitalisation between 28 and 35 days after vaccination [24]. In a study conducted in England in people aged 70 years and over, ChAdOx1 nCoV-19 was found to be 80% effective against hospitalisation after a single dose [25].

Multiple factors may have contributed to lower estimates observed in our study compared to VE estimates from other observational studies. Firstly, given that ChAdOx1 nCoV-19 (Covishield SII) was specifically indicated for use in the elderly, bias due to comorbidities, independent of the effect of age, may have been a fundamental cause of underestimation of effectiveness. Furthermore, the roll-out of ChAdOx1 nCoV-19 (Covishield SII) 3 weeks after BBIBP-CorV (Sinopharm), offered less time for follow-up of the ChAdOx1 nCoV-19 (Covishield SII) group over the study period. Secondly, the cautious approach in Seychelles during the first wave of the pandemic, that emphasised hospitalisation of COVID-19 cases (Fig 1) particularly the elderly and those with existing comorbidities, even when this was not necessarily indicated (i.e. low-threshold for admission) may have significantly underestimated VE for ChAdOx1 nCoV-19 (Covishield SII).

Finally, low VE estimates may also reflect lower effectiveness of ChAdOx1 nCoV-19 against the Beta VOC, as suggested by efficacy trials conducted in South Africa [26,27]. This is further supported by immunogenicity studies demonstrating a reduction in neutralising titres against the Beta variant after vaccination with ChAdOx1 nCoV-19 [27,28].

The strengths of our study include its population-based nature and the evaluation of two different vaccine types in a single population, enhancing the comparability of vaccine performance. Temporal bias, from changing public health and social measures (PHSM) and testing strategies over time, was minimised by adjusting for calendar months; whilst adjustments for age and gender reduced the potential risk of bias from prioritised groups for vaccination. A dataset linked by national identification numbers compiled from various national databases reduced the risk of recall bias and data on VOC obtained from genomic surveillance was validated by external laboratories, giving confidence in inferred patterns observed.

However, several important limitations exist. Despite adjustments for age, gender and calendar month, important covariates such as risk of exposure, comorbidities, prior infection with COVID-19 and socioeconomic status, were not included in the analysis. Exclusion of 1704 individuals due to missing or unconfirmed vaccination statuses and the relatively few hospitalisations and/or deaths included in our analysis as a result, may have impacted on the precision of our VE estimates. Other outcomes of interest such as infection, ICU admission, casualty admission, in addition to maternal and neonatal outcomes, were also not considered. While our study duration of 5 months provided valuable insight into VE over an extended timeframe, a longer follow-up duration is necessary to determine waning and effectiveness against new variant.

It is noteworthy, that by the end of the study period, Seychelles had achieved a remarkable milestone in vaccination coverage with over 70% population had received 2 doses of either vaccine. The attainment of such high vaccination coverage suggests for the potential establishment of a level of herd immunity, whereby a sufficiently large proportion of individuals within the community are immune to disease which could plausibly contribute for the relatively low numbers of hospitalization and deaths observed, minimizing the incidence of severe COVID-19 outcomes during the study period.

In conclusion, our results provide strong evidence of a high level of protection against hospitalisations and/or death with two doses of either BBIBP CorV (Sinopharm) or ChAdOx1 nCoV-19 (Covishield SII) vaccines within the context of Beta and Delta variant transmission in Seychelles. Our findings lend support for the use of a single dose of BBIBP CorV (Sinopharm) for protection against hospitalisation and/or death, in settings where vaccine supply and delivery may be constrained. Nevertheless, evidence points to two doses being preferable to boost and sustain protection against severe COVID-19. A single dose of ChAdOx1 nCoV19 (Covishield SII) was found to be inadequate in protecting against severe outcomes although this should be interpreted with caution given study limitations described. Evidence from our study supports maximising the uptake of two doses of ChAdOx1 nCoV (SII Covishield), particularly in older age groups, to ensure adequate protection.

Finally, considerations of vaccine access, supply and delivery, aside, our findings support the introduction of multiple vaccine types in a single population for better protection against co-circulation of multiple SARS-Co-V-2 variants. The results of this study are likely to be generalisable to other SIDS with similar population characteristics.

## Supporting information

S1 Fig Temporal distribution of circulating variants in Seychelles. (TIF)

S1 Table Comparison of characteristics of analysis sample to sample with missing vaccination status. (TIF)

S2 Table Comparison of estimates of vaccine effectiveness using Cox proportional hazards models versus modified Poisson regression. (TIF)

S3 Table Vaccine effectiveness (VE) against hospitalization/death by vaccine type defining individuals with missing vaccination status as unvaccinated. (TIF)
