# Supplementary figures and images for: Effectiveness of ChAdOx1 nCoV-19 and BBIBP-CorV vaccines against COVID-19-associated hospitalisation and death in the Seychelles infected adult population
Source: PLoS One. 2024 Apr 5;19(4):e0299747. doi: 10.1371/journal.pone.0299747 (PMC10997067; doi:10.1371/journal.pone.0299747)

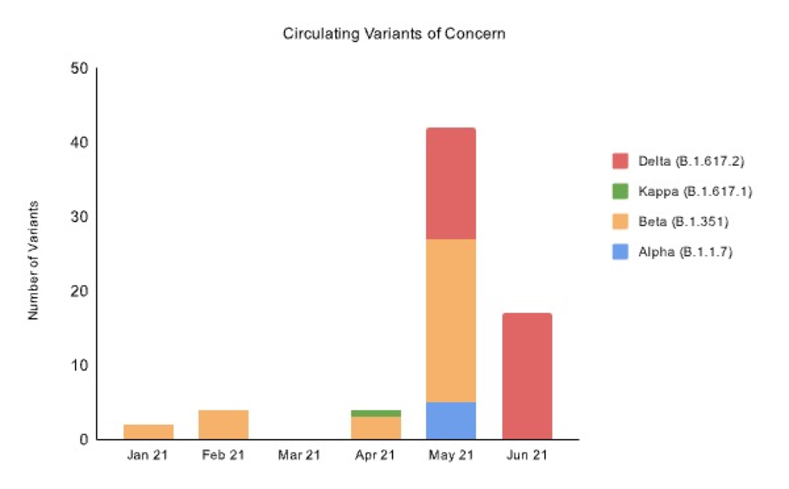

Supplement: S1 Fig — (TIF) [file pone.0299747.s001.tif]

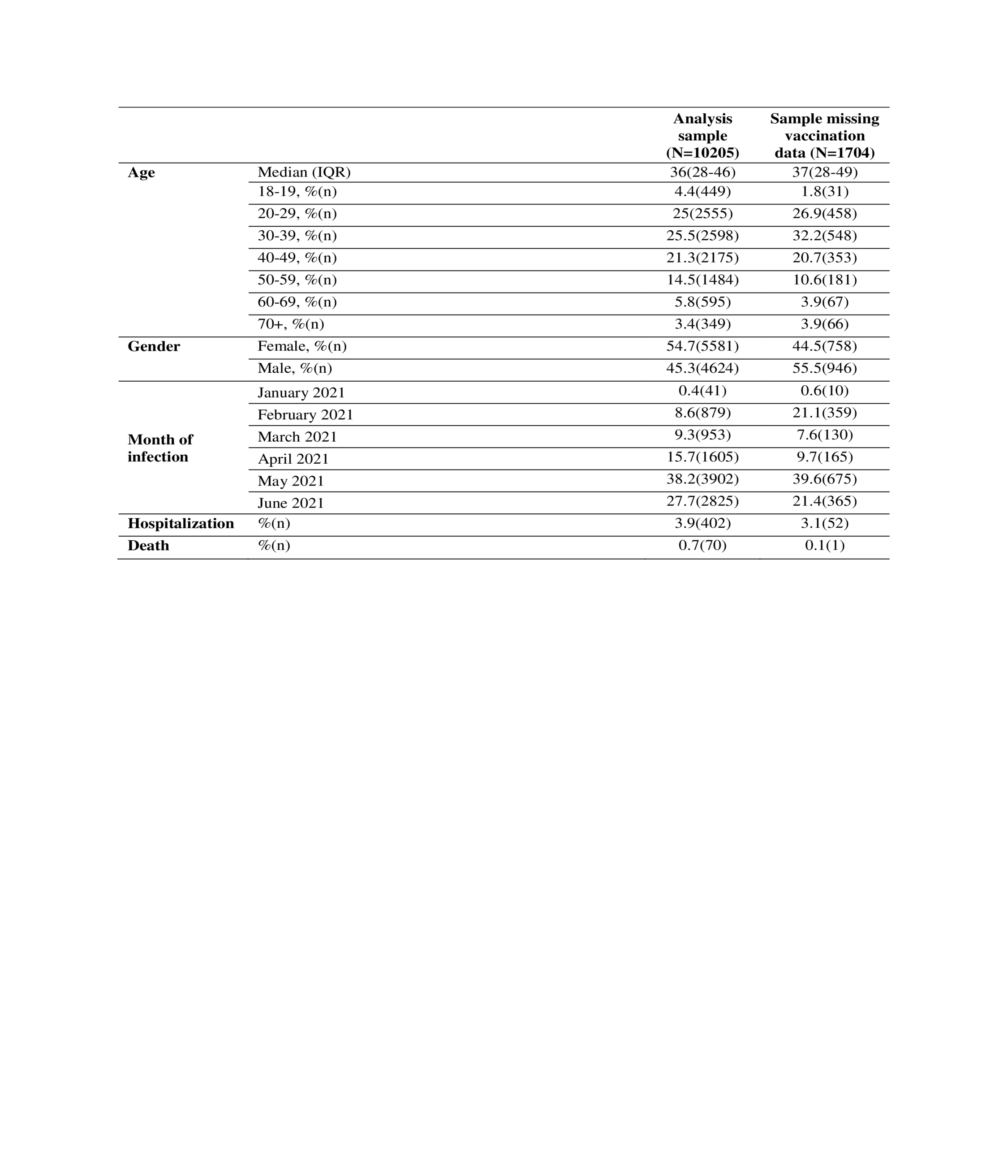

Supplement: S1 Table — (TIF) [file pone.0299747.s002.tif]

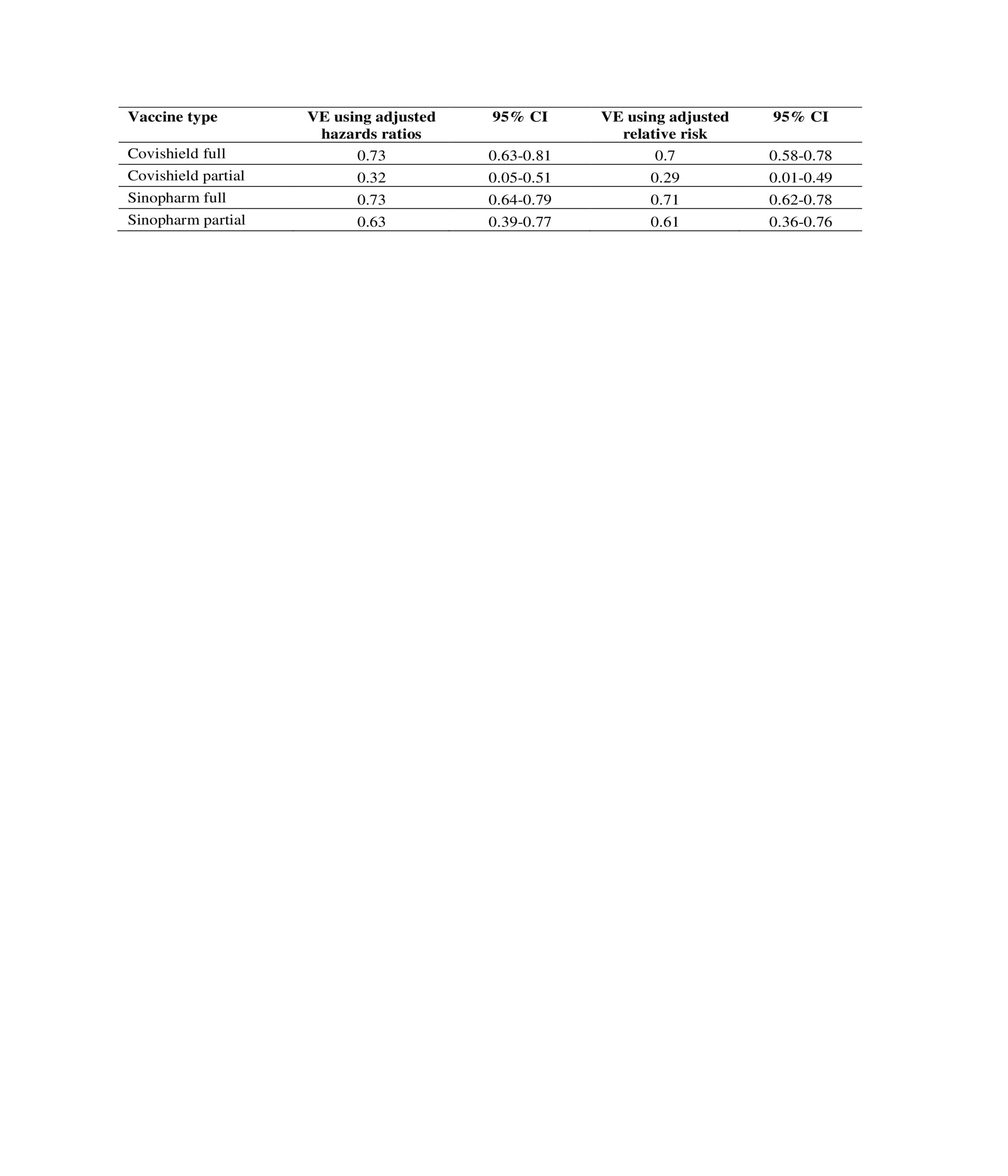

Supplement: S2 Table — (TIF) [file pone.0299747.s003.tif]

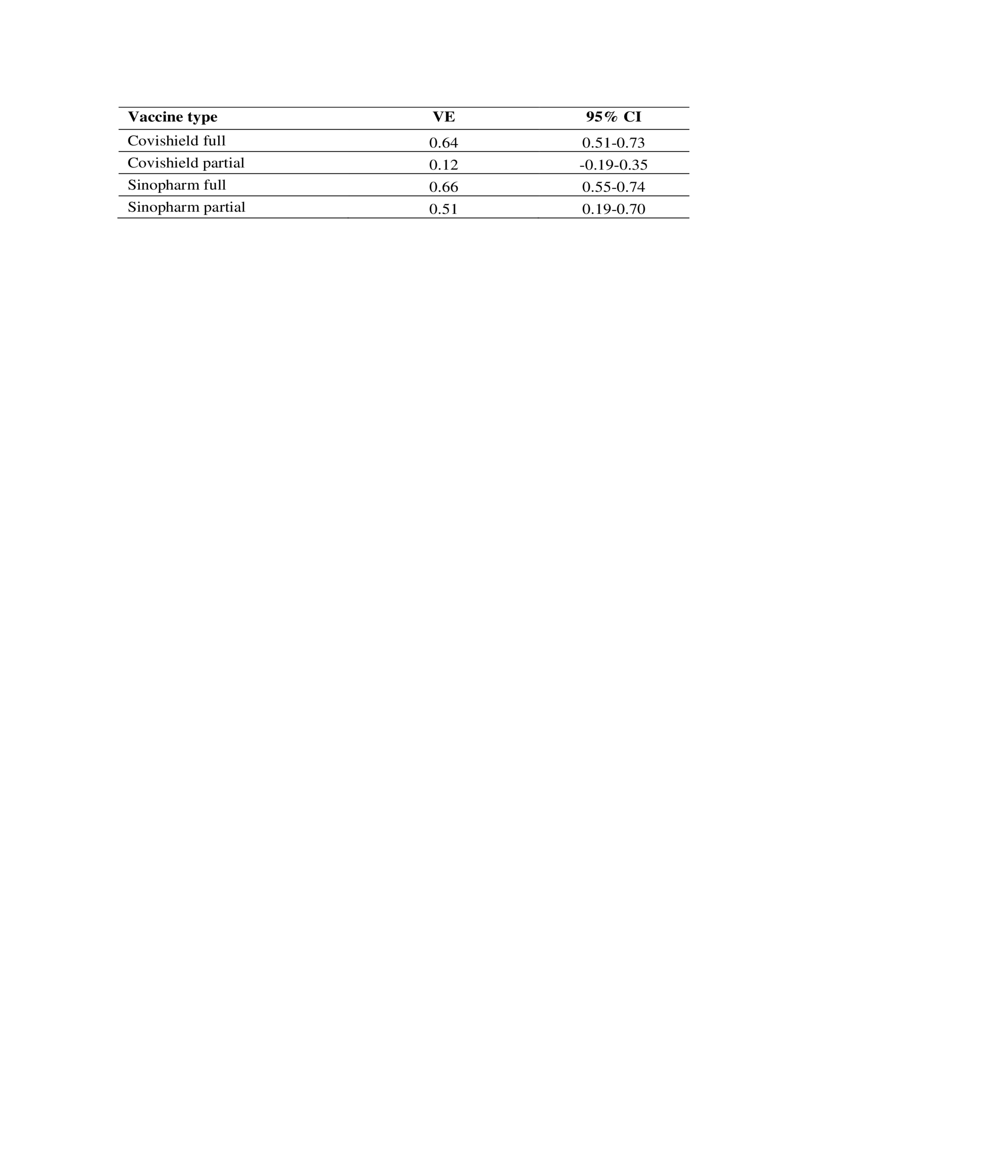

Supplement: S3 Table — (TIF) [file pone.0299747.s004.tif]
